# Medical management of non-obstructive azoospermia: A systematic review

**DOI:** 10.1080/2090598X.2021.1956233

**Published:** 2021-07-24

**Authors:** Mohammad H. Alkandari, Armand Zini

**Affiliations:** Division of Urology, Department of Surgery, McGill University, Montreal, Quebec, Canada

**Keywords:** Non-obstructive azoospermia, testicular sperm extraction, testicular failure, medical management, non-surgical management

## Abstract

While most men with non-obstructive azoospermia (NOA) are not amenable to medical treatment, some men can be treated effectively with hormonal therapy, prior to considering surgery. In some cases, hormonal therapy alone can treat NOA, without the need for surgery. In other cases, correction of a potential hormonal imbalance can enhance the chances of success of surgical sperm retrieval (SSR), with either conventional or microdissection testicular sperm extraction. Abnormal testicular function and low androgen levels can result from a primary dysfunction, a medical or surgical condition, or from an exogenous factor, and should be managed prior to more invasive interventions. Even men with normal androgen levels may benefit from hormonal therapy before sperm retrieval. Moreover, SSR may cause testicular injury and aggravate the pre-existing situation. If surgical extraction of sperm fails, it leaves the patients with less satisfactory options, like donor sperm or adoption. Therefore, it is the role of the infertility specialist to be vigilant and identify reversible causes of NOA, such as hormonal imbalance, prior to considering surgery. In the present paper we will systematically review the literature and highlight the available conventional medical regimens, as well as experimental ones.

**Abbreviations**: ART: assisted reproductive technology; CAH: congenital adrenal hyperplasia; EAU: European Association of Urology; hCG: human chorionic gonadotrophin; HH: hypogonadotrophic hypogonadism; hMG: human menopausal gonadotrophin; IUI: intrauterine insemination; micro-TESE: microdissection testicular sperm extraction; NOA: non-obstructive azoospermia; OR: odds ratio; SCO: Sertoli-cell only; SERM: selective oestrogen receptor modulator; SRR: sperm retrieval rate; SSC: spermatogonia stem cell; TART: testicular adrenal rest tumour; WMD: weighted mean difference

## Introduction

It is estimated that men are responsible for about half of couples’ infertility, and non-obstructive azoospermia (NOA) is present in 10–15% of the male infertility cases [1,2]. NOA represents the most severe form of male infertility, and its treatment remains challenging. Couples with NOA can be managed by sperm retrieval (with careful surgical dissection of the testicle, preferably microscopically) to harvest testicular spermatozoa, followed by assisted reproductive technology (ART). Alternatively, these men may be treated medically. The medical management of NOA is the topic of this review.

Understanding testicular structure and the underlying pathophysiology of NOA is a key element in the development of therapeutic modalities. The testis is composed of multiple cell types. The two cell types important for maintenance of spermatogenesis are the Sertoli and Leydig cells. While the former is an important supporting cell for developing germ cells in the testis, the latter produces testosterone, a key hormone in the maintenance of spermatogenesis [1]. Factors such as advancing age or gonadotoxin exposure will favour germ cell apoptosis and abnormal spermatogenesis.

## Methods

A systematic search of the National Library of Medicine (PubMed), Ovid Medline, EMBASE classic and EMBASE databases was performed in January 2021. This was done using the following Medical Subject Headings (MeSH) terms: ((‘medical management’[Mesh]) OR (‘non-surgical management’[Mesh])) AND ((‘non-obstructive azoospermia’[Mesh] OR (‘testicular sperm extraction’)). A subsequent search in relevant articles bibliographies, and reviews was conducted. Non-duplicate titles were screened. Full-text articles were included in the present review. Inclusion criteria comprised the use of non-surgical management in male human patients with NOA, either as a solitary intervention, or prior to surgical sperm retrieval (SSR). All treatment regimens were considered. Studies not available in English, or not respecting the inclusion criteria were excluded.

## Results

**Figure 1.**
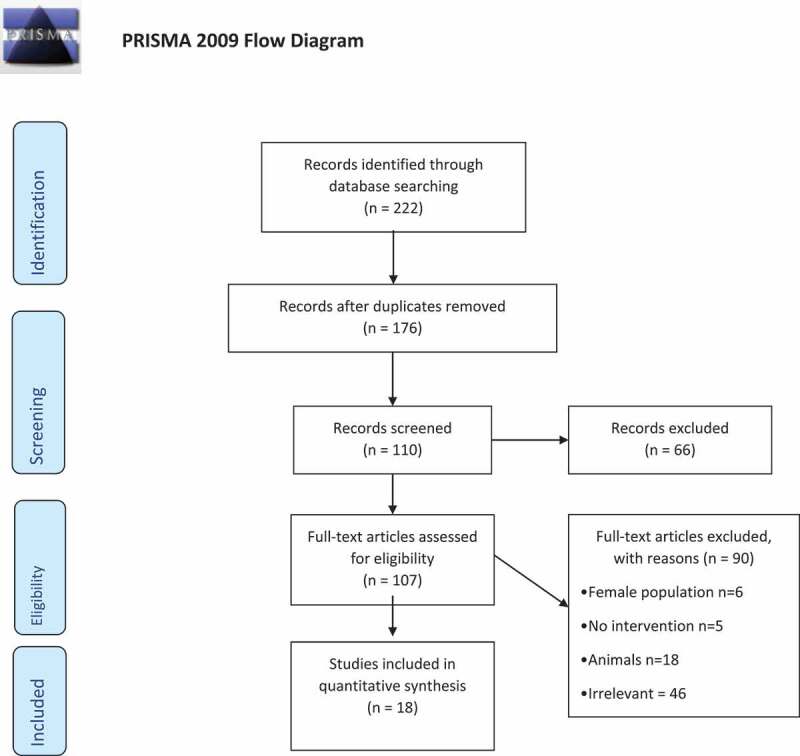
Systematic search strategy conducted in adherence to the Preferred Reporting Items for Systematic Reviews and Meta-Analyses (PRISMA) guidelines

A flow diagram of the literature search is presented in Figure 1. The previously described data search resulted in 222 articles. Duplicated publications were removed, 176 articles screened by title and abstract. Initially, 66 were excluded, because they did not follow the inclusion criteria. From the remaining 110 articles, three were abstracts only. The remaining 107 papers underwent a full-manuscript eligibility check, 18 (17%) were reserved for the purpose of the present review. A total of 90 studies were eliminated, six because they included female population only, there was no intervention in five, diet modification in two, surgical intervention in 13, and 18 were animal studies. However, most of the excluded articles, 64, reported findings out of the spectrum of the current research or were poorly constructed.

From the 18 remaining articles, three investigated medical treatment in men with NOA. Of those, there was a review, a descriptive clinical study and a comparative study. The descriptive study included 32 patients only but showed sperm recovery in ~50% of NOA men after tamoxifen. The comparative study was multicentric with a relatively large sample size (*n* = 612) and had a 2a level of evidence. It described a new protocol using clomiphene, human chorionic gonadotrophin (hCG) and human menopausal gonadotrophin (hMG) and compared them to a control group. The intervention led to the presence of sperm in the ejaculate in 11%, and sperm recovered surgically in 57% of the remainder. Another three articles focussed on medical optimisation prior to SSR. Of them, there were two systematic reviews and a retrospective study. One systematic review’s objective was to determine the best surgical technique in patients with NOA. The other one studied the optimisation of men with NOA prior to microdissection testicular sperm extraction (micro-TESE). The retrospective study focussed on the role of testicular biopsy in the diagnosis of azoospermic men. They concluded that biopsies can be avoided, as the vast majority of men with NOA (89%) have higher FSH levels (FSH >7.6 mIU/mL) and smaller testicular size (long axis <4.7 cm) when compared to obstructive cases. Two other reviews were recruited for the current publication, one described the general assessment of infertility in men, and the other one described the management of testicular adrenal rest tumours (TARTs)-related azoospermia. The latter review documented contradicting results on the ability of glucocorticoid therapy to regain fertility in those patients. However, they recommended sperm cryopreservation as a preventive method. A single prospective study discussed the different aetiologies of male infertility. The remaining 10 discussed different hormonal regimens to overcome male infertility. Among them, there were a single meta-analysis, three clinical trials, two reviews, two prospective, one comparative and retrospective articles. The meta-analysis focussed on idiopathic male infertility associated with abnormal semen parameters, other than NOA. Effect estimates were pooled using the Peto method with a fixed-effect model. It documented that using anti-oestrogens as an empirical therapy significantly increased the spontaneous pregnancy rate (odds ratio [OR] 2.42, 95% CI 1.47–3.94; *P* < 0.001), as well as both sperm concentration (weighted mean difference [WMD] 5.24, 95% CI 2.12–88.37, *P* = 0.001) and percentage sperm motility (WMD 4.55, 95% CI 0.73–8.37; *P* = 0.03) when compared to control. Furthermore, both FSH (WMD 4.19, 95% CI 2.05–6.34; *P* < 0.001) and testosterone (WMD 54.59, 95% CI 15.92–93.27; *P* = 0.006) were significantly raised as well. A clinical trial looked retrospectively at the effect of hormonal therapy in males with hypogonadotrophic hypogonadism (HH). They found a significant increase in testicular volume when hCG (50%) or combined hCG/hMG (75.7%) treatments were used, unlike testosterone. They also showed significant improvement in spermatogenesis with the same groups. The second clinical trial included 76 infertile males with idiopathic HH and tested the effect and predictors of success of prescribing GnRH on them. Pulsatile GnRH was proven beneficial on reaching adult values for testicular size (92%), FSH level (96%), and sperm in the ejaculate (100%) when there was no history of absent prior pubertal development. The last clinical trial in this group was multicentric and included 42 patients with NOA. They studied the administration of clomiphene and showed that 64.3% produced sperm in the ejaculate (mean of 3.8 million/mL), and 100% had successful surgical extraction of sperm. The remaining articles in this group showed some promising and other conflicting results on medical management. Unfortunately, apart from the single meta-analysis study, none of the recruited articles were randomised. Therefore, we added two international guidelines to the pool of this review, the combined AUA/American Society for Reproductive Medicine (ASRM) on male infertility management, and the European Association of Urology (EAU) on male hypogonadism.

## Aetiology of NOA

In a large prospective study of >1700 infertile men, the aetiology of azoospermia is estimated to be caused by genetic factors (26%), obstruction (26%), congenital factors (14%), oncological factors (8%), secondary hypogonadism (5%)’ and other factors (3%) [3]. The cause of NOA is primarily congenital with 15% of cases being acquired. In some cases, including those resulting from congenital abnormalities, NOA can be managed medically.

The NOA aetiologies amenable to medical therapy, include the following: 1) HH, 2) excess androgens or steroids secondary to an exogenous source or androgen-producing tumour, or 3) hypergonadotrophic hypogonadism or primary testicular failure. Primary testicular failure is often irreversible (genetic defect, chemotherapy, post-traumatic, post-torsion) but it may be caused by potentially reversible factors (e.g. varicocele, infection).

## Evaluation of NOA

Men screened on initial evaluation and found to be azoospermic should be further evaluated with a complete reproductive history and physical examination. The evaluation is designed to identify potential causes of azoospermia. A physical examination will specifically help evaluate testicular volume and consistency and confirm presence or absence of vasa and varicocele. A repeat semen analysis is often requested to confirm the diagnosis of azoospermia. A hormonal evaluation will help differentiate between primary (hypergonadotrophic hypogonadism) and secondary testicular failure (HH). Men with hypergonadotrophic hypogonadism generally present with testicular atrophy and have an elevated serum FSH on hormonal profiling. Men with idiopathic hypergonadotrophic hypogonadism should also be offered genetic evaluation with a karyotype and Y chromosome microdeletion analysis. Genetic evaluation is required to uncover the cause of infertility and to counsel couples regarding the risk of possible transmission of genetic defects to the offspring.

## Management of NOA (according to aetiology)

The three NOA aetiologies that are associated with hormonal imbalance and may potentially benefit from medical management are: 1) HH, 2) excess androgens or steroids and 3) hypergonadotrophic hypogonadism.

A well-studied group is patients with HH. The condition is defined as decreased testicular production of testosterone secondary to decreased production of LH from the pituitary gland. These men will have low LH and FSH, as well as, low testosterone levels on their hormonal profile. It can be associated with congenital syndromes, such as Kallmann syndrome, Prader–Willi syndrome and Laurence–Moon syndrome, or results from insults to pituitary gland like direct trauma or radiotherapy. The rest of the HH cases are usually idiopathic.

Hypogonadotrophic hypogonadism is managed medically with a high success rate [4], but specific medical treatment is only applied when the couple wishes to conceive [5]. Exogenous testosterone will correct the low serum testosterone levels in these men, but this treatment will lead to a negative feedback on the pituitary and hypothalamus resulting in low intra-testicular testosterone production and impaired spermatogenesis. The preferred medication for men wishing to maintain normal intra-testicular testosterone production and re-establish spermatogenesis is hCG. hCG directly stimulates the LH receptors on the Leydig cells. Recombinant FSH or hMG can be added to enhance spermatogenesis. The hCG/hMG combination has shown superiority over solo treatment with hCG regarding spermatogenesis, 60% vs 40%, respectively [6]. In the case of FSH (or a FSH analogue), it is started after normalising testosterone levels with hCG [7]. According to the EAU guidelines, in order to reach a normal testosterone level in blood, an hCG dose of 1500–5000 IU is given intramuscularly or subcutaneously twice weekly, while a FSH regimen of 150 IU is given three-times weekly intramuscularly or subcutaneously [8]. If this regimen is continued for 6 months, the success rate (defined as sperm in the ejaculate) is ~80% [1]. However, to maximise the chances of achieving spermatogenesis, the course of therapy might be extended for up to 2 years [9]. An alternative regimen, with a similar success rate, albeit more expensive, is the administration of GnRH in a pulsatile manner [10]. The most convenient route for GnRH administration is a bi-hourly dose regimen of 5–20 µg over 12–24 months via a subcutaneous pump infusion.

A second form of hormonal imbalance that can lead to azoospermia is an excess in circulating androgens as a result of either exogenous intake or overproduction by tumour. These men will have low LH and FSH but normal to high testosterone levels on their hormonal profile. The cessation of exogenous anabolic hormones will usually promote the testicles to regain their function spontaneously within a year. On the other hand, when the source is endogenous, like in congenital adrenal hyperplasia (CAH), elevated level of adrenocorticotrophic hormone (ACTH) increases the adrenal’s sex hormone (androstenedione) production, resulting in negative feedback on pituitary and hypothalamus, thus suppressing testicular androgens production. The popular CAH therapy with glucocorticoids usually protects the testes and resumes sperm production. If this regimen resulted in HH and azoospermia, then the treatment is the same as described above for HH. However, the most common cause of infertility in patients with CAH is the development of TARTs in ~40% of patients [11]. The development of TARTs is mainly associated with the advancement of CAH severity. Here lies the importance of close follow-up of those patients before developing TARTs and recommending sperm cryopreservation if available.

The third category that also is generally not amenable to medical management, when surgically reversible causes like varicocele are excluded, is primary or intrinsic testicular failure. These men will generally have high LH and FSH with normal to low testosterone levels on their hormonal profile. This condition can be progressive and potentially rescuable if detected early. On physical examination, the smaller and softer testes are indicative of abnormal spermatogenesis and testicular failure [12,13]. The predominant remaining cell type in the majority of these men are Leydig and Sertoli cells. Both cell types rely on the pituitary’s gonadotrophins (LH and FSH) to produce testosterone and sperm. In the case of hypergonadotrophic hypogonadism, despite the elevated level of LH and FSH, testosterone is generally decreased. How could that happen? Frequently, it is secondary to peripheral conversion of testosterone to oestrogen, thus reducing the intra-testicular testosterone level, a prerequisite for sperm production. Studies have shown that men with NOA and hypergonadotrophic hypogonadism have significantly lower testosterone:oestrogen ratio than fertile men [10]. Therefore, interruption of the peripheral conversion of testosterone to oestrogen is expected to elevate circulating testosterone, as well as the levels of intra-testicular testosterone.

Common ways of increasing intra-testicular testosterone levels in men with NOA and hypergonadotrophic hypogonadism are by use of aromatase inhibitors (steroidal and non-steroidal) and anti-oestrogens. Aromatase inhibitors block aromatase enzyme, which converts testosterone to oestrogen in peripheral tissues. A comparative study on 140 infertile men with an abnormal testosterone:oestrogen ratio showed that both groups of aromatase inhibitors significantly improved that ratio but were never successful in producing sperm in the ejaculate [14]. The same study group also reached the conclusion that patients might benefit from this treatment if they are undergoing a sperm retrieval surgery. Another study showed similar results when men with low testosterone and either a low testosterone:oestrogen ratio or failed clomiphene were prescribed daily anastrozole (non-steroidal aromatase inhibitor) [15]. Although, none of these men had sperm in the ejaculate after treatment, 73% underwent successful micro-TESE after anastrozole.

With respect to anti-oestrogens or selective oestrogen receptor modulators (SERMs), such as clomiphene citrate and tamoxifen, these appear to have different effects on men with NOA. These drugs inhibit oestrogen receptors at the level of the pituitary, thus promoting the secretion of both LH and FSH, which will enhance testicular function. Oestrogen modulators, or even hCG, can be used as a solitary treatment or in combination to correct testosterone levels [8]. The AUA guidelines indicate that prescribing these hormones for idiopathic infertility with normal testosterone levels may be useful as well. In a study of patients with NOA with hypospermatogenesis or maturation arrest, but not Sertoli-cell only (SCO), 64% produced sperm in the ejaculate and the remainder had successful sperm retrieval intervention after use of clomiphene citrate [16]. The dose was adjusted according to testosterone level, with a starting dose of 50 mg every other day for 2 weeks. Tamoxifen had also showed similar effects in a meta-analysis [17]. In fact, tamoxifen showed positive results in several types of testicular dysfunction, including SCO. Overall, 19% showed sperm in the ejaculate (none in SCO), and 50% showed sperm on the testicular biopsy [18]. A common therapeutic scheme is 10–30 mg daily for 3–6 months. Nevertheless, in cases of SERM failure gonadotrophins may be prescribed, as earlier described for HH [19].

In couples wishing to proceed to micro-TESE, optimisation of testicular function by hormonal therapy can be attempted. Despite the limited literature supporting this approach, gonadotrophin or anti-oestrogen therapy may improve the sperm retrieval rate (SRR) in men with NOA [6,20]. Hussein et al. [21] evaluated the effect of clomiphene citrate ± hCG, or hCG/hMG combination in men with NOA. Using this treatment, they aimed to raise the FSH level at 1.5-times baseline, and a testosterone of 600–800 ng/dL. They observed that if that regimen successfully increased the levels of FSH and testosterone, they obtained a high SRR at micro-TESE.

In conclusion, although surgical extraction of sperm with ART may result in pregnancy, it neither reverses the infertility pathology nor treats the underlying cause of NOA and it may result in deterioration of the testicular condition. Medical therapy is available for men with NOA, particularly those with a hormonal deficiency. These couples may benefit from the medical intervention either through production of sperm in the ejaculate, or indirectly, by improving SRRs at the time of surgical dissection. Succeeding to convert azoospermia to severe oligozoospermia might not be sufficient to induce a natural pregnancy but may allow for successful intrauterine insemination (IUI) or intracytoplasmic sperm injection pregnancy and avoid testicular dissection. Thus, a thorough medical and endocrinological evaluation is essential before proceeding to a more invasive procedure.

## Recent findings

The main challenge in any medical problem is treating a disease with unknown aetiology. In the case of idiopathic NOA, we have begun to slowly unravel some of the aetiologies of this condition. Novel treatment options for this specific population of infertile men face many ethical and technical barriers and, therefore, remain experimental. Some of the breakthrough approaches for patients with idiopathic NOA are spermatogonial stem cell (SSC) transplantation, *in vitro* spermatogenesis, and gene therapy [22]. SSCs have the potential to produce spermatozoa when activated by Sertoli cells products. Other stem cells which can be transplanted are embryonic and adult pluripotent cells. The potential drawback with germ cell transplantation, as with gene therapy, is the risk of cancer development. Gene therapy has been studied in humans with diseases caused by specific gene defects. Unfortunately, the genetic causes of NOA have not been fully uncovered, which makes genetic intervention more challenging. These new technologies have been used successfully in animal models but have yet to be applied to humans [22].

## Summary

Although combined surgical sperm extraction and ART may enable couples with NOA to have a child, they do not treat the underlying pathology. Medical therapy for carefully selected patients might achieve the same results, without exposing both partners to invasive interventions. Moreover, if medical management fails to allow couples to conceive naturally, they may benefit from medical therapy in one of two ways; by improving surgical sperm extraction rates or by producing sperm in the ejaculate adequate for less invasive ART interventions, like IUI. Therefore, a thorough medical evaluation is valuable for all infertile men.
